# Intellectual Impairment in Patients with Newly Diagnosed HIV Infection in Southwestern Nigeria

**DOI:** 10.1155/2015/185891

**Published:** 2015-07-29

**Authors:** Taofiki A. Sunmonu, Johann Sellner, Olubunmi A. Ogunrin, Frank A. Imarhiagbe, Morenikeji A. Komolafe, Olusegun T. Afolabi, Olayinka S. Ilesanmi, Fatai Olanrewaju, Benedicta Y. Oladimeji

**Affiliations:** ^1^Neurology Unit, Department of Medicine, Federal Medical Center, Owo 341, Ondo State, Nigeria; ^2^Department of Neurology, Christian-Doppler-Klinik, Paracelsus Medical University, 5026 Salzburg, Austria; ^3^Department of Neurology, Klinikum rechts der Isar, Technische Universität München, 81675 München, Germany; ^4^Neurology Unit, Department Medicine, University of Benin Teaching Hospital, Benin 300, Nigeria; ^5^Department of Community Health, Obafemi Awolowo University Teaching Hospitals Complex, Ile-Ife 220, Osun State, Nigeria; ^6^Department of Community Health, Obafemi Awolowo University, Ile-Ife 220, Osun State, Nigeria; ^7^Department of Community Health, Federal Medical Centre, Owo 341, Ondo State, Nigeria; ^8^Department of Dermatology, Obafemi Awolowo University Teaching Hospitals Complex, Ile-Ife 220, Osun State, Nigeria; ^9^Clinical Psychology Unit, Department of Mental Health, Obafemi Awolowo University, Ile-Ife 220, Osun State, Nigeria

## Abstract

Neurocognitive impairment is a detrimental complication of HIV infection. Here, we characterized the intellectual performance of patients with newly diagnosed HIV infection in southwestern Nigeria. We conducted a prospective study at Owo Federal Medical Center by using the adapted Wechsler Adult Intelligence Scale (WAIS). The raw scores were converted to standardized scores (*z*-scores) and correlated with clinical and laboratory findings. Fifty-eight HIV positive patients were recruited; 72% were in WHO stages 3 and 4. We detected a high rate of intellectual impairment in HIV positive patients and controls (63.8% and 10%, resp.; *P* < 0.001). HIV positive patients performed worse throughout the subtests of both verbal and performance intelligence quotients. Presence of opportunistic infections was associated with worse performance in the similarities and digit symbol tests and performance and full scale scores. Lower body weight correlated with poor performance in different WAIS subtests. The high rate of advanced disease stage warrants measures aimed at earlier diagnosis and treatment. Assessment of neurocognitive performance at diagnosis may offer the opportunity to improve functioning in daily life and counteract disease progression.

## 1. Introduction

Human immunodeficiency virus (HIV) and acquired immunodeficiency syndrome (AIDS) continue to take their toll on the life of the patients and their caregivers. Sub-Saharan Africa is the most heavily affected region in the world with estimated 1.9 of the 2.5 million new HIV infections worldwide in 2011 [1]. Most of the fatal courses remain in the context of late diagnosis of HIV infection and delayed initiation of antiretroviral therapy (ART) [2, 3].

A major concern for public health is the occurrence of HIV-associated neurocognitive disorders (HAND) [4]. The manifestations range from asymptomatic or mild neurocognitive impairment to HIV-associated dementia. Intellectual impairment in HIV infection may cause unemployment, susceptibility for systemic disease, and impaired quality of life [5, 6]. Gender and drug-related risk behavior were identified as risk factors for HIV-associated neurocognitive impairment and an inverse correlation with adherence to ART was reported [7, 8]. This is detrimental, as compliance with ART can achieve almost a normal life span and physical and emotional health. In resource-poor settings, however, diagnosis as well as initiation and maintenance of ART have been associated with major challenges. Notably, undetected cognitive impairment can increase the risk of poor medication adherence and impair social and academic functioning [9].

Nigeria has the second largest number of inhabitants living with HIV worldwide, and a prevalence rate of 3.6% was calculated for 2011 [2]. For Nigeria as for Sub-Saharan Africa, there is paucity of information on neurocognitive functioning in individuals with newly diagnosed HIV infection. The first step was done by Royal III and coworkers, who established a neuropsychological testing battery and reported a rate of 29% for abnormal performance among 60 HIV positive ART-naïve Nigerian patients [10]. None of the previous studies in the region used the Wechsler Adult Intelligence Scale (WAIS) so far. The test is designed to measure intelligence-related domains in adults and older adolescents [11]. The main advantage of the WAIS series is the comprehensive nature of the tests and includes different subtest of verbal intelligence quotient (IQ) and performance IQ. Results of both scores are summarized as full scale IQ.

The aim of this study was to determine pattern and severity of intellectual impairment as well as risk factors among patients with newly diagnosed HIV infection in southwestern Nigeria.

## 2. Material and Methods

### 2.1. Ethics Approval

The Ethical Committee of Owo Federal Medical Center gave approval for the study. Each participant signed the consent form and the study was conducted in accordance with the Declaration of Helsinki (1964).

### 2.2. Setting

The study was performed at Owo Federal Medical Centre. This tertiary care facility was appointed by the federal government as a HIV competence center for southwestern Nigeria. The major aim is the provision of free access to medical care and therapies including ART and laboratory investigations.

### 2.3. Study Population

This prospective trial recruited patients with newly diagnosed HIV infection. The patients either presented to the outpatient clinic or were newly admitted to the medical ward. The inclusion criteria were as follows: age greater than 16 years, completion of at least primary school education, as some of the test items require the patient to be literate and proficient in English language to understand the test items, and positive HIV serostatus. Patients already on ART were excluded. Further exclusion criteria were the presence of behavioral problems that would interfere with comprehension of the test items, current use of psychoactive drugs/alcohol abuse, and history of other neurological disorders not attributable to HIV infection such as stroke, Parkinson's disease, or epilepsy. A psychiatric disorder or current intake of antipsychotic drugs, anemia (hematocrit < 20%), severe functional impairment (Karnofsky performance score < 50), or severe medical illness that would interfere with the ability to undergo neuropsychological evaluation were further exclusion criteria.

The control group consisted of seronegative patients attending the outpatient clinic of the hospital. Applicable exclusion criteria were used for the seronegative controls.

### 2.4. Clinical Examination

The patients were staged for HIV infection using the World Health Organization (WHO) rating system. A questionnaire was used for sociodemographic data and medical history. We assessed the presence of opportunistic infections in HIV infected patients. Pulmonary tuberculosis was diagnosed with a chest X-ray and sputum examination. Empirical clinical response to antitoxoplasmosis drugs was taken as evidence of toxoplasmosis infection.

### 2.5. Laboratory Testing

Blood samples were collected for packed cell volume determination (hematocrit) and CD4 lymphocyte count was assessed using automated flow cytometry.

### 2.6. Adapted Wechsler Adult Intelligence Scale (WAIS)

The intellectual performance was assessed in English with the adapted WAIS, version of 1955. This test was validated in a cohort of Nigeria Technical College students and was previously used to study Nigerian patients with epilepsy [12, 13]. The adaptation of the WAIS was based on previous work by D. E. Itsuokor and modified to suit the Nigerian population (unpublished Ph.D. thesis, University of Ife, Nigeria, 1981). The items modified were as follows: information items (1, 6, 7, 9, 10, 14, 17, 19, 20, 21, 24, 25, 27, 28), comprehension items (7, 11, 12, 13, 14), arithmetic items (3, 4, 5, 7, 9, 10, 13), vocabulary items (4, 17, 37, 38, 40), and a picture completion item (13). The most recent WAIS IV was not utilized because it has not been validated for the Nigerian population.

The subcategories of verbal IQ include the verbal comprehension index (CI) and the working memory index (WMI), whereas perceptual organization index (POI) and processing speed index (PSI) assess the performance IQ. Initially, the raw scores were recorded. The mean and standard deviation of the controls on each WAIS variable were calculated and this served as the basis for converting the raw scores of all the entire 108 participants to standard (*z*) scores for each of the WAIS subtests.

### 2.7. Statistical Analysis

Data were analyzed by SPSS version 21.0 software (SPSS Inc., Chicago, IL). Student's *t*-test was used to compare continuous variables and Fisher exact test (chi-square) to compare categorical variables. Linear regression was used to evaluate association of cognitive impairment with clinical and laboratory variables. Probability levels < 0.05 were taken as significant.

## 3. Results

### 3.1. Patients and Demographics

We enrolled 58 HIV positive patients (men 58.6%) and 50 controls (men 64%). Ethnical background was Yoruba in the majority (76%) and 9% were Igbos and 2% Hausa. The remainder were from minority tribes. Details of the sociodemographics, WHO stage, and comorbidities are shown in Tables 1 and 2. There was a one-year higher average education in the HIV-control group. Statistical analysis did not reveal differences among the groups.

### 3.2. Laboratory Findings

Half of the HIV positive patients had mild to moderate anemia. Sixty % had a CD4 count <200 cells/UL. Opportunistic infections were present in WHO HIV stages 3 and 4 in 50% and 72%, respectively.

### 3.3. Intellectual Performance

Neurocognitive impairment as evidenced by a full scale *z*-score below one SD was present in 63.8% of the HIV infected patients and 10% of the controls (*P* < 0.001). Further details are shown in Table 4. HIV infected patients performed worse compared to the controls on WAIS in different domains including verbal and performance scores (*P* < 0.001). We found significantly lower mean *z*-scores for majority of the WAIS subtests. A large Cohen's effect size (*d*) was found in all analyses. Details are shown in Table 3.

### 3.4. Relationship of Gender and Intellectual Impairment

No gender-related neurocognitive differences were detected. Intellectual impairment was present in 100% of females and 97% of males in the HIV infected patients while intellectual dysfunction was present in the 61.1% of females and 59.4% of males in the control group.

### 3.5. Analysis of Intellectual Performance and Clinical/Laboratory Variables

*Anemia*. The PCV did not correlate with the verbal score, performance score, and full scale score. In the WAIS subtests, there was a correlation for the performance on the picture arrangement subtest and PCV (*P* < 0.05); further details are shown in supplements 1–4 in the Supplementary Material available online at http://dx.doi.org/10.1155/2015/185891.

*CD4+ Cell Count*. There was no statistically significant association between the CD4 count and the patients' performance on the WAIS subtests, verbal scores, performance scores, and full scale scores.

*Presence of Opportunistic Infections (OIs)*. HIV positive patients with opportunistic infections performed significantly worse than those without OIs on similarities and digit symbol test, which was reflected by lower performance score and full scale scores (*P* < 0.05). Subgroup analysis of the intellectual function of HIV positive patients with opportunistic infections showed that there was no significant difference among the various opportunistic infection categories (*P* > 0.05). 

*WHO HIV Stage*. There were no significant associations between the WAIS subtests mean scores, verbal score, performance score, full scale score, and WHO HIV stage of the patients. 

*Weight*. Weight was analyzed separately for men and women. Men in the weight range 60–69 kg performed better than in the other weight categories on similarities, pictures completion, performance score, and full scale score subtests of the WAIS (*P* < 0.05). Women in the weight range 60–69 kg performed significantly better on comprehension and similarities subtests compared to women in lower weight categories (*P* < 0.05). There was no significant association between the weight of patients with HIV infection and other WAIS subtests.

## 4. Discussion

Here, we aimed to characterize intellectual functioning in yet ART-naïve adult patients with newly diagnosed HIV infection. The study is unique as the WAIS was used and the majority of the patients were in advanced WHO stages. We report a high prevalence of intellectual impairment in this cohort and confirm and propose several risk factors for impaired intellectual functioning but could not identify a typical neurocognitive domain altered with HIV infection.

Commonly used instruments for the assessment of neurocognitive impairment in patients with HIV infection include the HIV dementia scale (HDS), the International HIV Dementia Scale (IHDS), Community Screening Interview for Dementia, and Minimental State Examination (MMSE) [4, 14, 15]. Computer-delivered cognitive assessment batteries such as Cogstate (http://www.cogstate.com/) and Fepsy (http://www.fepsy.com/), as well as the Montreal Cognitive Assessment (MCA), are further test batteries that have been used [16]. In this context, the prevalence rate of neurocognitive impairment in HIV infected patient differs substantially among studies due to different methodological approach and study population [17]. Choice of normative data may also influence prevalence estimates. Here, we used sample standardized scores to reduce this bias. Yet, the high rate of intellectual impairment (63.8%) is more than that of a pivotal Nigeria study (54%) using IHDS [18]. In a South-Asian population, the prevalence of HAND was found to be 22.7% using MCA and IHDS [19]. A recent Ugandan study found severe NCI to be present in 27% of patients with HIV infection [20]. Using an abbreviated test battery, a prevalence rate of 19% for neurocognitive impairment was detected early in the course of HIV infection in the USA [21]. Of note, a recent survey in USA identified neurocognitive impairment in 47% even in the era of ART [22]. The high rate of intellectual impairment identified in our study can be mostly traced back to advanced disease stages in the majority of our patients. A predominant affection of the fronto-striato-thalamo-cortical systems was reported in neuropathological studies [23, 24]. Subsequently, the main impairment would be expected for executive functions (e.g., planning), memory, and psychomotor speed, with relative sparing of basic language and visuoconstructive skills. The WAIS is not focused on the assessment of cognitive functions including episodic memory and executive functions. However, our study did disclose reduced scores throughout the WAIS but did not identify such a pattern. Yet, a recent study from Cameroon revealed that AIDS patients performed worse than non-AIDS HIV infected patients on cognitive function test including performances on WAIS III symbol search tests [25].

There might be other factors apart from acquired brain injury from HIV infection and related conditions that might have contributed to the poor intellectual performance. This may for instance be related to impaired premorbid intellectual functioning. There have been reports that baseline IQ and higher cognitive reserve provided more predictive information than the CD4 count and plasma HIV RNA levels [26]. Thus, the limitation that premorbid IQ levels were not available in our study needs to be taken into account. Another factor might be the age at which HIV was acquired. In this regard, a recent study showed that HIV infection during brain development in youth and adulthood has more profound effect on neurocognitive disturbances related to frontostriatal circuits [27]. Intrinsic factors related to test performance need to be considered when studying a Sub-Saharan population compared to European or American counterparts using the WAIS. Though English is the official language in Nigeria, the country is a multilingual society with different local languages being spoken. It is possible that variability in the degree to which these indigenous languages are spoken may affect the test result. Subsequently, we recruited participants that were able to read and write in English language and 91% of the participants had greater or equal to 11 years of education.

An increased rate of neurocognitive impairment is not only found with advanced stages of HIV infection. Previous studies had reported a variety of risk factors including alcohol and substance abuse, lower nadir CD4 counts, cardiovascular/metabolic diseases, psychiatric disorders, hepatitis C virus coinfection, host genetic factors, virus subtype, anemia, and opportunistic infections [6, 17]. Beforehand, viral load determination was not available in this study because of the costs. We focused on the association of CD4 counts, WHO stage, anemia, and body weight. The markers of immune compromise such as low CD4 cell counts and high HIV RNA levels are associated with diffuse cortical atrophy, disease progression, and higher risk of death [28]. In this study, there was no significant association between CD4 count and intellectual performance. This may be due to smaller proportion of patients with higher CD4 count. In analogy, the WHO clinical stages were not associated with the intellectual performances of the patients, most likely related to the preponderance for more advanced disease stages. Anemia was shown to be associated with impaired cognitive function and daily living activities in elderly people [28]. Anemia may not have influenced intellectual function in our cohort because patients with severe anemia and poor Karnofsky performance were excluded. Weight loss continues to be a common problem in the era of HAART and is associated with reduced quality of life. Notably, weight loss has also been associated with disturbed neurocognitive function even in non-HIV infected patients. A study showed a moderate impairment of memory and motor skills among HIV infection men with lower body weight [29]. This keeps with our finding that men with lower body weight performed worse on the picture completion subtest, performance score, and full scale. In this study, lower body weight in women was associated with poorer performance on verbal items tests such as comprehension and similarities subtests.

There is paucity of the literature on the effect of OIs on intellectual functions in patients with HIV infection. In this study, patients with OIs had poorer performance on similarities and digit symbol test, performance scores, and full scale scores when compared to the patients without OIs. We did not identify specific OIs to be associated with a specific pattern of impairment. A more pronounced effect of OIs was reported by Wang et al. where CNS toxoplasmosis and cryptococcal meningitis were associated with HIV-associated dementia [30].

## 5. Conclusions 

Large scale campaigns have been launched to ensure early diagnosis and treatment of HIV infection and associated conditions as these measures were shown to improve mortality, morbidity, and subsequent quality of life. This study, however, demonstrated that many patients are still not diagnosed and treated at an early disease stage. A subsequent high rate of intellectual impairment and comorbidities is present in this cohort. The impact of psychiatric disease was not evaluated as only patients with minor psychiatric symptoms were included. Additional cause for structural brain damage and CNS infections could have been present in the cohort but was not evaluated due to lack of resources for brain imaging and laboratory testing. Further studies may also take the impact of HIV-associated cancer into account. Taken together, the findings of this study have potential implications for collaborative diagnostic and therapeutic efforts to be further undertaken in order to reduce the rate and consequences of unfavorable comorbidities associated with HIV infection.

## Supplementary Material

Supplement 1: Laboratory variables and intellectual performance.
Supplement 2: Opportunistic infections and intellectual performance.
Supplement 3: Clinical variables and intellectual performance.

## Figures and Tables

**Table 1:** Sociodemographics.

Variables	HIV	Controls	*P*
	*N* = 58	*N* = 50	
Sex	*N* (%)	*N* (%)	
Men	34 (59)	32 (64)	ns
Women	24 (41)	18 (36)	
Duration of education			
Mean ± SD (years)	12.4 ± 3.3	13.3 ± 2.1	ns
Age (years)			
Mean ± SD	35.9 ± 8.0	35.4 ± 11.5	ns

ns = not significant.

SD = standard deviation.

**Table 2:** Clinical and laboratory finding in HIV positive patients.

	*N*	(%)
Packed cell volume (PCV)		
20–29	28	50.0
30–39	25	44.6
40–50	3	5.4
CD4 cell count (cells/*µ*L)		
>200	35	60.3
200–349	14	24.1
350–499	5	8.6
≥500	4	6.9
Presence of opportunistic infections		
PTB	16	27.6
Oral candidiasis	4	6.9
CNS toxoplasmosis	2	3.5
PTB + oral candidiasis	6	10.3
Herpes skin infection + PTB	1	1.7
None	29	50.0
WHO HIV stage		
1	5	8.6
2	11	19.0
3	36	62.0
4	6	10.3
Weight (kg)		
30–39	6	11.1
40–49	16	29.6
50–59	21	38.9
60–69	9	16.7
≥70	2	3.7

**Table 3:** Analysis of WAIS means *z*-scores in HIV positive patients and controls.

Tests	HIV+ (*N* = 58)	Controls (*N* = 50)	*t*	*d*	*P*
	Means ± SD	Means ± SD			
WAIS (verbal)					
Information	−0.77 ± 0.62	0.00 ± 1.00	−4.913	0.94	<0.001
Comprehension	−0.73 ± 0.90	0.00 ± 1.00	−4.010	0.77	<0.001
Arithmetic	−0.57 ± 0.59	0.00 ± 1.00	−3.634	0.71	0.001
Similarities	−0.85 ± 0.62	0.00 ± 1.00	−4.857	1.04	<0.001
Digit spam	−0.88 ± 0.82	0.00 ± 1.00	−5.666	0.94	<0.001
Vocabulary	−0.55 ± 0.61	0.00 ± 1.00	−3.529	0.68	0.001
WAIS (performance)					
Digit symbol	−1.32 ± 0.95	0.00 ± 1.00	−7.037	1.36	<0.001
Picture completion	−1.02 ± 0.47	0.00 ± 1.00	−6.962	1.34	<0.001
Block design	−1.50 ± 1.06	0.00 ± 1.00	−7.526	1.45	<0.001
Picture arrangement	−0.86 ± 1.67	−0.01 ± 1.00	−5.291	1.01	<0.001
Object assembly	−1.05 ± 0.69	0.00 ± 1.00	−6.422	1.24	<0.001
Verbal score	−0.74 ± 0.54	0.00 ± 1.00	−4.891	0.94	<0.001
Performance score	−1.57 ± 0.77	0.00 ± 1.00	−9.234	1.78	<0.001
Full scale score	−1.22 ± 0.60	−0.01 ± 1.00	−7.750	1.49	<0.001

*t* = Student's *t*-test value.

*d* = Cohen's effect sizes value.

0.2: small effect sizes.

0.5: medium effect sizes.

≥0.8: large effect sizes.

**Table 4:** Gender and the intellectual function in HIV positive patients and controls using the full scale (Fs) mean *z*-scores.

	Total number of subjects with Fs mean *z*-scores <1 SD	Men	Women
	*N* (%)	*N* (%)	*N* (%)
HIV+	37/58 (63.8)	22/34 (65)	15/24 (62.5)
Control	5/50 (10.0)	2/32 (6.3)	3/18 (16.7)

Fs mean *z*-score <0.00 = impaired Fs mean *z*-score.
