# Supplementary material for: Intellectual Impairment in Patients with Newly Diagnosed HIV Infection in Southwestern Nigeria
Source: Biomed Res Int. 2015 Jul 29;2015:185891. doi: 10.1155/2015/185891 (PMC4532809; doi:10.1155/2015/185891)
Supplement: Supplementary file 1 — Supplement 1: Laboratory variables and intellectual performance. Supplement 2: Opportunistic infections and intellectual performance. Supplement 3: Clinical variables and intellectual performance. [file 185891.f1.pdf]

Supplement 1. Laboratory variables and intellectual performance in HIV positive patients using WAIS mean z - scores

Variables	N	Info	Compr	Arith	Simi	Dig Span	Vocab	Dig Symb	Pic Compl	Blk Desg	Pic Arran	Obj Ass	Verb Score	Perf Score	FS Score
PCV(%)															
20–29	28	- 0.71	- 0.82	- 0.66	-1.04	- 0.99	-0.61	- 1.48	- 1.01	- 1.71	- 0.62	- 1.06	- 0.81	-1.61	- 1.31
>30	28	- 0.82	- 0.62	- 0.48	-0.63	- 0.74	-0.48	- 1.17	- 1.02	- 1.30	- 0.09	- 1.01	- 0.65	-1.52	- 1.12
t		0.708	- 0.830	- 1.099	-1.900	- 1.600	-0.830	- 1.201	0.072	- 1.466	2.714	- 0.279	- 1.090	-0.423	- 1.198
p		0.482	0.410	0.277	0.063	0.115	0.410	0.235	0.943	0.149	0.009	0.781	0.281	0.667	0.236
CD_4 Cell Count (Cells / uL)															
<200	35	-0.70	-0.70	-0.55	-0.92	-0.84	-0.55	-1.45	-1.00	-1.59	-0.83	-0.88	-0.73	-1.63	-1.26
200 – 349	14	-0.80	-0.84	-0.52	-0.81	-1.08	-0.43	-1.28	-1.07	-1.45	-1.09	-1.27	-1.71	-1.57	-1.18
350 – 499	5	-1.03	-0.75	-0.66	-0.93	-0.39	-0.83	-0.56	-0.90	-1.35	-0.58	-1.34	-0.91	-1.21	-1.12
> 500	4	-0.77	-0.70	-0.77	-0.33	-1.06	-0.68	-1.29	-1.28	-0.98	-0.73	-1.41	-0.79	-1.51	-1.22
F		0.660	0.092	0.241	0.644	2.003	0.586	1.338	0.595	0.441	0.900	1.949	0.181	0.432	0.107
p		0.580	0.964	0.867	0.590	0.124	0.627	0.272	0.621	0.725	0.448	0.133	0.909	0.731	0.956

Supplement 2: Opportunistic infections and intellectual performance in HIV+ patients using WAIS mean z-scores

Variables	N	Info	Compr	Arith	Simi	Dig Span	Vocab	Dig Symb	Pic Compl	Blk Desig	Pic Arrag	Obj Ass	Verb Score	Perf Score	Fs Score
Presence of Opportunistic Infections															
Yes	29	-0.85	-0.81	-0.63	-1.18	-0.93	-0.60	-1.71	-1.05	-1.75	-0.83	-1.03	-0.85	-1.79	-1.41
No	29	-0.69	-0.65	-0.50	-0.53	-0.82	-0.51	-0.93	-0.99	-1.25	-0.90	-1.07	-0.64	-1.35	-1.02
t		-0.987	-0.684	-0.848	-3.250	-0.735	-0.599	-3.392	-0.428	-1.830	0.422	0.201	-1.500	-2.264	-2.562
P		0.328	0.497	0.400	0.002	0.465	0.552	0.001	0.670	0.073	0.675	0.841	0.139	0.027	0.013
Type of Opportunistic Infections															
PTB	16	-0.91	-0.68	-0.69	-1.25	-1.04	-0.73	-1.80	-1.20	-1.85	-0.86	-1.16	-0.92	-1.85	-1.53
Oral Cand	4	-0.51	-0.57	-0.36	-0.54	-0.76	-0.22	-1.53	-0.58	-1.47	-1.09	-0.74	-0.45	-1.76	-1.11
CNS Toxo	2	-0.51	-1.83	-1.05	-1.63	-1.16	-0.22	-1.93	-1.16	-1.34	-1.09	-1.13	-0.89	-1.89	-1.43
PTB + Oral Candidiasis	6	-0.99	-0.91	-0.59	-1.30	-0.76	-0.58	-1.53	-0.94	-1.75	-0.61	-0.92	-0.88	-1.66	-1.32
Skin Herpes + PTB	1	-1.23	-1.31	-0.23	-0.98	-0.55	-0.90	-1.73	-0.90	-1.84	-0.01	-0.67	-1.04	-1.58	-1.38
F		0.568	0.734	0.522	-1.091	0.497	0.816	0.217	2.173	0.210	0.705	0.427	0.575	0.105	0.425
p		0.688	0.578	0.720	0.383	0.738	0.528	0.926	0.103	0.930	0.596	0.788	0.683	0.980	0.789

Supplement 3: Clinical variables and Intellectual Performance in HIV+ patients using WAIS mean z- Scores

Variables	N	Info	Compr	Arith	Simi	Dig Span	Vocab	Dig Symb	Pic Comp	Blk Desig	Pic Arrag	Obj Ass	Verb Score	Perf Score	Fs Score
WHO HIV Stage															
1	5	-0.42	-0.69	-0.61	-0.20	-1.12	-0.27	-1.22	-1.05	-1.15	-1.30	-1.05	-0.48	-1.53	-1.02
2	11	-0.78	-0.68	-0.50	-0.60	-0.63	-0.62	-0.70	-1.18	-0.99	-0.73	-1.11	-0.70	-1.17	-1.13
3	36	-0.89	-0.78	-0.58	-0.99	-0.94	-0.65	-1.50	-0.99	-1.76	-0.89	-1.05	-0.84	-1.70	-1.30
4	6	-0.37	-0.62	-0.59	-1.01	-0.76	-0.10	-1.49	-0.94	-1.11	-0.61	-0.92	-0.46	-1.57	-1.02
F		1.922	0.075	0.059	1.920	1.213	1.931	2.179	0.539	2.175	1.200	0.097	1.399	1.315	0.726
P		0.137	0.973	0.981	0.137	0.314	0.135	0.101	0.657	0.102	0.318	0.962	0.253	0.279	0.541
Weight (kg) Men															
40 – 49	9	-0.77	-0.29	-0.50	-0.30	-0.96	-0.41	-1.43	-0.76	-1.02	-0.65	-0.81	-0.54	-1.30	-0.94
50 – 59	16	-0.75	-0.64	-0.53	-1.17	-0.96	-0.72	-2.00	-1.22	-1.75	-1.13	-1.30	-0.84	-2.10	-1.50
60 – 69	4	-0.47	-0.27	-0.29	-0.38	-1.37	-0.31	-0.68	-0.52	-1.23	-0.73	-0.27	-0.45	-0.93	-0.71
≥70	2	-0.92	-0.52	-0.23	-0.65	-0.96	-0.75	-1.47	-1.28	-0.86	-1.09	-1.01	-0.49	-1.54	-1.85
F		0.576	1.315	0.236	2.366	0.490	1.062	2.618	4.797	1.135	1.797	2.248	1.214	4.081	3.740
P		0.636	0.290	0.870	0.093	0.692	0.381	0.071	0.008	0.352	0.171	0.106	0.324	0.016	0.023
Weight (kg) Women															
30 – 39	6	-0.94	-1.49	-0.55	-0.83	-1.03	-0.66	-1.18	-1.24	-2.17	-0.85	-1.03	-0.96	-1.73	-1.40
40 – 49	7	-1.01	-1.61	-0.66	-1.53	-0.73	-0.74	-1.08	-1.12	-1.70	-0.62	-1.15	-1.10	-1.49	-1.37
50 – 59	5	-0.80	-1.31	-0.72	-1.19	-0.47	-0.62	-0.68	-0.80	-1.06	-0.87	-1.15	-0.89	-1.18	-1.09
60 – 69	5	-1.06	-0.62	-0.72	-0.20	-0.63	-0.53	-0.35	-1.00	-1.25	-0.44	-1.34	-0.69	-1.06	-0.92
F		0.267	3.280	0.108	5.518	1.156	0.192	1.221	0.797	1.331	0.370	0.271	1.023	1.089	1.100
P		0.848	0.043	0.954	0.007	0.352	0.900	0.329	0.510	0.294	0.776	0.846	0.405	0.378	0.374

Info – information
Compr – Comprehension
Arith – Arithmetic
Simi – Similarities
Verb Score – Verbal score

Dig Span – Digit Span
Vocab – Vocabulary
Dig Symb – Digit Symbol
Pic Compl – Picture Completion
Perf Score – Performance Score

Blk Desig – Block Design
Pic Arrang – Picture Arrangement
Obj Ass – Object Assembly
FS – Full Scale Score
